# Preoperative endoscopic titanium clip placement facilitates intraoperative localization of early-stage esophageal cancer or severe dysplasia

**DOI:** 10.1186/s12957-017-1188-2

**Published:** 2017-08-02

**Authors:** Lei Tan, Juan Feng, Qin Zhao, Ping Chen, Guotao Yang

**Affiliations:** 1 Department of Thoracic Surgery, QILU Hospital, Shandong University, 44# Wenhua Xi Road, Jinan, 250012 People’s Republic of China; 2 Department of Thoracic Surgery, Central Hospital of Taian, Taian, 271000 People’s Republic of China; 3 Department of Surgical Oncology, Central Hospital of Taian, Taian, 271000 People’s Republic of China; 4 Department of Gastroenterology, Central Hospital of Taian, Taian, 271000 People’s Republic of China

**Keywords:** Esophageal cancer, Dysplasia, Endoscopy, Titanium clip

## Abstract

**Background:**

Accurate intraoperative localization of esophageal lesions is essential for successful surgical resection. We tested whether preoperative endoscopic placement of titanium clips could facilitate intraoperative localization of early-stage esophageal cancer or severe dysplasia.

**Methods:**

A prospective randomized clinical trial was performed between May 2012 and July 2014. All enrolled patients received preoperative endoscopy and esophageal endoscopic ultrasound, as well as pathological study on the biopsy specimen, to confirm early stage esophageal cancer or severe dysplasia. One day before the surgical operation, patients in the experimental group received the preoperative endoscopic titanium labeling of esophageal lesions. Then, during the surgical operation, palpitation of titanium clips was used to localize the lesions in these patients. In patients in the control group, palpitation of nodules or esophageal wall mucosal thickening, together with the consideration of the results from preoperative endoscopic and ultrasound studies, was applied to estimate the location of the esophageal lesions. Study outcomes included the proportions of patients having successful intraoperative pre-resection lesion localization, post-esophagectomy lesion visualization, negative upper surgical margin, change of surgical approaches, and positive postoperative pathological diagnosis.

**Results:**

A total of 27 patients were enrolled into the study, with 14 in the experimental group and 13 in the control group. Compared to the patients in the control group, a higher proportion of patients in the experimental group had statistically significant successful intraoperative esophageal lesion localization (100 versus 15.3% in the experimental versus control group).

**Conclusions:**

Preoperative endoscopic titanium clip placement could facilitate intraoperative localization of early-stage esophageal cancer or severe dysplasia.

**Trial registration:**

Current study was registered in Chinese Clinical Trial Registry and World Health Organization International Clinical Trials Registry Platform, ChiCTR-INR-17010949. Registered 22 March 2017, retrospectively registered.

## Background

Esophageal cancer is the sixth most common cause of cancer death worldwide [1]. With the wide application of endoscopic examination, an increasing number of patients with early-stage esophageal cancer was diagnosed. The recommended treatment for early-stage esophageal cancer is surgical resection [2, 3]. Successful surgical resection depends on accurate localization of esophageal lesions. Traditionally, during the surgical operation, palpation of the esophageal wall, together with the consideration of the preoperative testing results from endoscopic or other imaging studies (barium x-ray, computed tomography, endoscopic ultrasound), was used to estimate the location of esophageal lesions [4, 5]. The success with this localization technique varies with operator’s experience. A better localization method is required in order to achieve satisfactory resection of early-stage esophageal cancer.

Recent years, different preoperative labelling methods, such as dye staining, tattooing, and metal clip, have been shown to facilitate intraoperative localization of gastrointestinal cancers [6–8]. However, there was a limited study on the preoperative labeling of esophageal lesion [9]. We performed a preliminary study to preoperatively apply titanium clips to early-stage esophageal lesions. We reported our study results here.

## Methods

### Study design

We conducted a prospective randomized clinical trial in an urban academic hospital between May 2012 and July 2014. The study protocol was approved by the hospital ethics committee and registered in Chinese Clinical Trial Registry and World Health Organization International Clinical Trials Registry Platform (ChiCTR-INR-17010949). All of the study participants signed the informed consent.

### Participant selection

The inclusion criteria were (1) patients with early-stage T1 esophageal squamous cell carcinoma or severe dysplasia detected by endoscopy and esophageal endoscopic ultrasound. The lesion was biopsied and confirmed by the pathological study; (2) scheduled for surgical resection by the treating physicians. The exclusion criteria were (1) history of thoracic surgery procedure which would make it difficult for the operation and (2) history of allergy to titanium.

### Study protocol

All patients received the preoperative endoscopy and esophageal endoscopic ultrasound examination. They were randomly assigned into either the experimental group or the control group [10]. One day before the surgical operation, patients in the experimental group received repeated endoscopic examinations. Once the esophageal lesion was localized, two titanium clips (EZ Clip, HX-610-135 L, Olympus, Japan) were placed on the upper and lower edges of the lesions. Then, 2 h after endoscopy, these patients underwent an upper gastrointestinal tract meglumine diatrizoate swallowing study in order to confirm the location of the titanium clips.

The Sweet surgical procedures were performed for all study patients by the same group of surgeons. All the surgical operations were performed through a left chest posterolateral incision. An esophagogastrostomic anastomosis below the aortic arch was applied for esophageal lesions localized >35 cm below the incisor. A supra-aortic arch esophagogastric anastomosis was used for esophageal lesions between 25 to 35 cm below the incisor.

During the surgical operation, the esophageal lesions were localized by palpation for the titanium clips in patients in the experimental group. For patients in the control group, palpation for nodules or thickened esophageal wall together with the consideration of preoperative endoscopy and ultrasound examination results was applied to estimate the location of the esophageal lesions. After localization of the esophageal lesions, esophagus was transected 5 cm above the upper border of the lesion. The lower segment of the esophagus, including proximal part of the stomach, was also transected. Then, an esophagogastrostomic or esophagogastric anastomosis was performed. The transected esophagus was examined intraluminally for lesions, such as mucosal erosions, ulcers, or nodules. If the lesions were <5 cm away from the upper resection margin of the esophagus, additional segment of the esophagus was removed to make sure that the distance between the lesions and resection margins was >5 cm. If the intrathoracic anastomosis could not ensure that the distance was >5 cm between the lesions and resection margins, a left neck incision would be added and the surgical approach would be changed to a subtotal esophagectomy through a left chest incision followed by a cervical esophagogastric anastomosis. The resected esophageal segments were sent for the pathological study.

### Study outcomes

Study outcomes included the proportions of patients having successful intraoperative pre-resection lesion localization, post-esophagectomy lesion localization, negative upper surgical margin, change of surgical approaches, and positive postoperative pathological diagnosis.

A successful intraoperative pre-resection lesion localization was obtained when the titanium clip was palpated in the experimental group, or a nodule or thickened esophageal wall was palpated in the control group. A successful post-esophagectomy lesion localization was the positive finding of either mucosal erosion, ulcer, or nodule when examining resected esophageal segment after the operation in both experimental and control groups. After surgical operation, all the resected esophageal segments were sent for pathological study. A positive postoperative pathologic diagnosis was defined when the postoperative pathological examination confirmed the existence of squamous cell carcinoma or severe dysplasia.

### Statistical analysis

Numerical variables were presented as mean ± standard deviation. Categorical variables were presented as percentages. Group comparisons were performed by chi-square analysis. Analyses were performed using SPSS (version 19.0). All statistical tests were two-sided with a significance (a) level of 0.05.

## Results

**Fig. 1:**
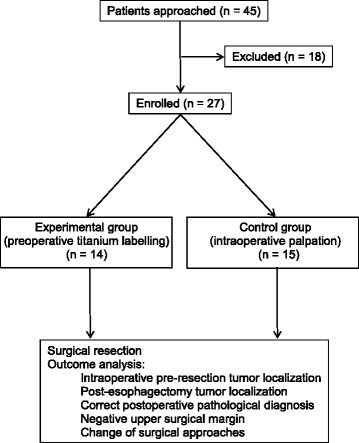
CONSORT diagram

**Fig. 2:**
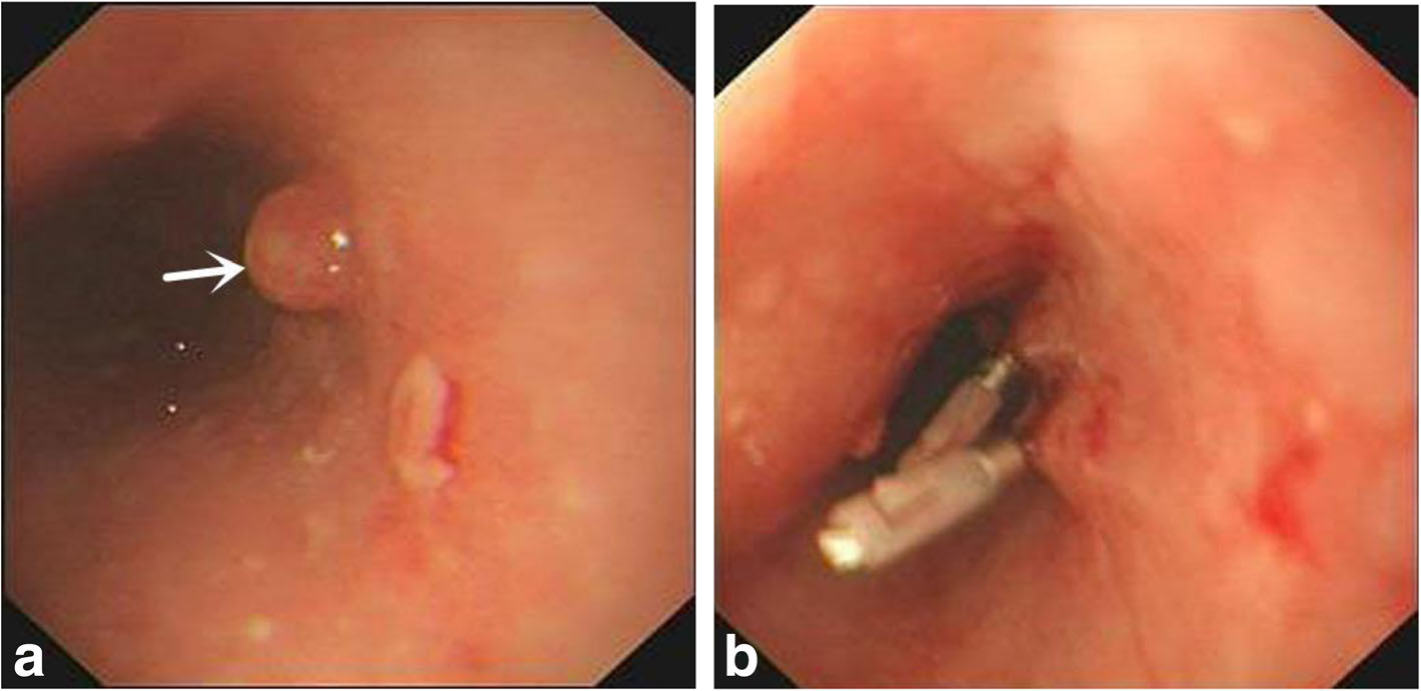
Preoperative endoscopic examination. **a** Esophageal lesions. **b** Titanium clips were applied to the esophageal lesions

**Fig. 3:**
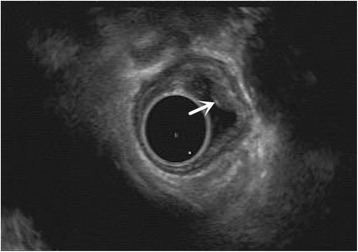
Endoscopic ultrasound examination shows an intramural subepithelial nodule

**Fig. 4:**
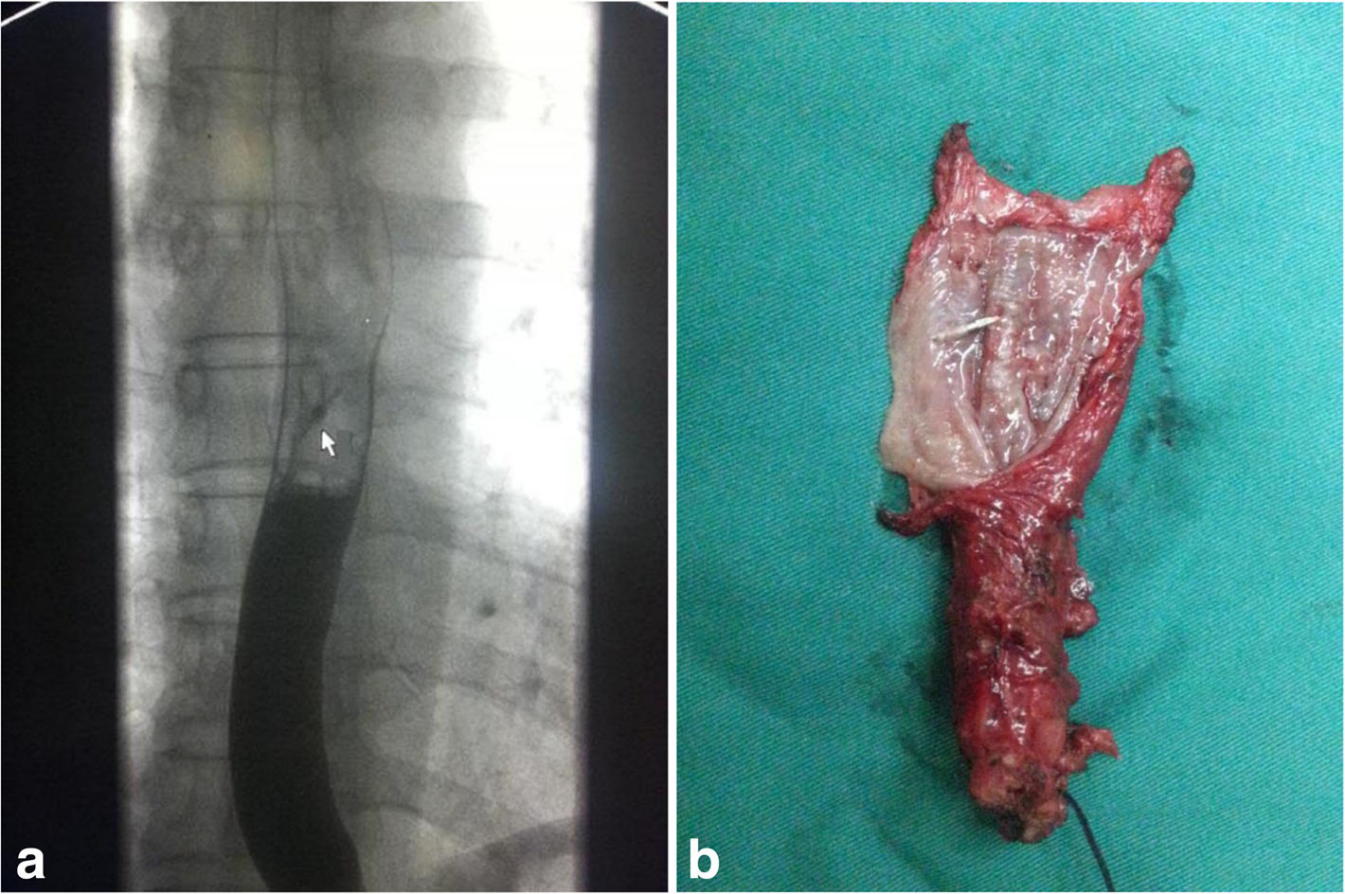
**a** A titanium clip (*arrow*) is shown in the upper gastrointestinal tract meglumine diatrizoate swallowing study. **b** A titanium clip is shown in the resected esophageal segment

A total 27 patients were enrolled into the study, with age 61.9 ± 5.7 years, and 66.7% (18) male patients (Fig. 1). Fourteen patients were in the experimental group and 13 patients were in the control group. Their baseline characteristics were listed in Table 1. Examples of esophageal lesions under endoscopic examination and placement of titanium clips were shown in Fig. 2a, b, respectively. Endoscopic ultrasound result was shown in Fig. 3. A titanium clip could be seen in a patient undergoing the upper gastrointestinal tract meglumine diatrizoate swallowing study (Fig. 4a). A titanium clip could be seen in a resected esophageal segment (Fig. 4b).

**Table 1:** Baseline characteristics for study participants (*N* = 27)

	Experimental group	Control group
	(*N* = 14)	(*N* = 13)
Age, year, mean ± SD	61.5 ± 6.1	62.3 ± 5.5
Gender		
Male, *N* (%)	10 (71.4%)	8 (61.5%)
Type of esophageal lesion, *N* (%)		
Squamous cell carcinoma	14 (100%)	11 (84.6%)
Severe dysplasia	0 (0%)	2 (15.4%)
Location of esophageal lesion, *N* (%)		
Middle segment	12 (85.7%)	8 (61.5%)
Lower segment	2 (14.3%)	5 (38.5%)
Tumor staging, *N* (%)		
T1a	1 (7.1%)	3 (23.1%)
T1b	13 (92.9%)	10 (76.9%)

Table 2 shows the comparisons of successful intraoperative pre-resection lesion localization, post-esophagectomy lesion localization, negative upper surgical margin, change of surgical approaches, and positive postoperative pathological diagnosis between two study groups. Compared to the control group, statistically significantly higher proportion of patients in the experimental group had successfully intraoperative pre-resection lesion localization.

**Table 2:** Outcome comparisons between two study groups

	Experimental group	Control group
	(*N* = 14)	(*N* = 13)
Intraoperative pre-resection lesion localization, *N* (%) *	14 (100%)	2 (15.4%)
Post-esophagectomy lesion localization, *N* (%)	14 (100%)	10 (76.9%)
Negative upper surgical margin, *N* (%)	14 (100%)	12 (92.3%)
Change of surgical approaches, *N* (%)	1 (7.1%)	2 (15.4%)
Positive postoperative pathological diagnosis, *N* (%)	13 (92.8%)	12 (92.3%)

*P < 0.05 between two groups

## Discussion

Our study showed that preoperative endoscopic titanium clip placements could facilitate intraoperative lesion localization in patients with early stage esophageal cancer or severe dysplasia.

The primary treatment for esophageal cancer is the surgical resection [2, 3]. Precise intraoperative cancer localization is important for successful surgical resection. Patients with esophageal cancer commonly received preoperative gastroscopy, endoscopic ultrasound, barium X-ray, or CT scans. Traditionally, intraoperative palpation of the esophagus at the approximate site identified from these preoperative examinations was used to estimate the location of the esophageal cancer during the surgical resection. However, the small size of early-stage esophageal cancer usually made the intraoperative palpation difficult. In our current study, the traditional intraoperative palpation only resulted in a 15.3% success rate to localize early-stage esophageal lesions. Preoperative titanium placement improved the success rate to 100%.

Currently, esophageal cancer resection mainly includes intrathoracic and cervical anastomosis [11]. Intrathoracic anastomosis is further divided into supra-aortic arch or infra-aortic arch approaches, which is usually determined by the location of the upper edge of the tumor, since a complete tumor removal requires resection margins at least 5 cm away from the edges of the tumor [12]. By preoperative marking of the edges of the tumor with titanium clips, it is easier to intraoperatively select an appropriate location of anastomosis based on a “safe” distance to the marker clips. In the experimental group, postoperative pathological examination showed that all of the 14 patients had a negative upper resection margin, indicating a high reliability of this method. One patient had surgical resection method changed in the experimental group due to the high position of esophageal lesion. An initially scheduled supra-aortic arch esophagogastric anastomosis was changed to cervical esophagogastric anastomosis in order to make sure the safe distance (>5 cm) between the lesion margin and resection site.

In the control group, the tumor location was estimated based on the preoperative imaging findings and intraoperative palpation of the esophageal wall. After the esophageal transection in this group of patients, the distance between the tumor edge and the upper resection margin of the esophagus was found too short and thus the anastomosis location had to be changed in two patients. In one of the two patients, the predetermined anastomosis below aortic arch was changed to a supra-aortic arch anastomosis, and in the other patient, the intrathoracic anastomosis was changed to a cervical anastomosis. This suggested that the intraoperative estimation of the tumor position based on the preoperative imaging findings and intraoperative palpation was not very accurate.

Our results showed that the postoperative pathological examination showed negative results in two patients tested (one patient in the experimental group and one in the control group). The possible reason might be that the primary lesion was small and was completed removed during the first endoscopic biopsy.

Due to rich lymphatic vessels in the esophageal mucosa, a jumping metastasis often occurs in patients with esophageal cancer [13]. Currently, the main approach to reduce the positive rate of resection margins is an intraoperative frozen section examination of esophageal resection margins [14, 15]. A study has shown that the accuracy of intraoperative frozen sectioning for margin evaluation was 93% and all errors of frozen section evaluation occurred at the proximal margin [7]. Therefore, in order to achieve a margin-negative resection, the distance from the resection margin to the tumor is very important. In the experimental group in the current study, the location of the anastomosis was selected after adequate distance was considered according to the location of titanium clips, ensuring the highest possible negative rate of resection margins. However, in the control group, the judgement for the distance from the resection margin to the tumor could be inaccurate due to the difficult intraoperative tumor localization, leading to an increased positive rate of resection margins (7.7%).

Limitations of the current study included single center and small sample size study. We also did not follow up these patients for their long-term outcomes.

## Conclusions

In summary, preoperative endoscopic titanium clip labelling could facilitate intraoperative lesion location in patients with early-stage esophageal cancer or severe dysplasia.
